# Functionalization of aminoalkylsilane-grafted cotton for antibacterial, thermal, and wettability properties

**DOI:** 10.1039/d2ra03214g

**Published:** 2022-07-21

**Authors:** Abeer Alassod, Mina Shahriari-khalaji, Yujie Wang, Andrew Balilonda, Mhd Firas Al Hinnawi, Shengyuan Yang

**Affiliations:** State Key Laboratory for Modification of Chemical Fibers and Polymer Materials, Shanghai “Belt & Road” Joint Laboratory of Advanced Fibers and Low-dimension Materials, College of Materials Science and Engineering, Donghua University Shanghai 201620 People's Republic of China cmseysy@dhu.edu.cn; Textile Industries Mechanical Engineering and Techniques Department, Faculty of Mechanical and Electrical Engineering, Damascus University Damascus Syria; Research Centre for Smart Wearable Technology, Institute of Textiles and Clothing, The Hong Kong Polytechnic University Hung Hom Kowloon 999077 Hong Kong P. R. China; Biomedical Engineering Department, Faculty of Mechanical and Electrical Engineering, Damascus University Damascus Syria

## Abstract

Multifunctional cotton fabrics are considered a significant challenge, hindering their commercialization through a scalable and eco-friendly method. The main drawbacks that limit their wide application are the lack of antibacterial activity, wettability, and being easily damaged by fire. Herein, we report a facile synthesis technique of superhydrophobic, flame resistant and antibacterial cotton fabric production using APTES agents to achieve all the above-mentioned properties. This study optimized the chemical grafting of aminoalkylsilane on the cotton surface with different reaction times and APTES concentrations to get the highest grafting content. Chemical characterization confirmed successful aminoalkylsilane grafting on the surface of cotton fabric. Subsequently, the antibacterial activity, wettability, and flame resistance properties of aminoalkylsilane grafted cotton fabric were accurately investigated. The obtained results showed that samples at 10 h reaction time with 14% APTES concentration indicated higher grafting content which showed high enhancement. Additionally, all produced aminoalkylsilane grafted cotton demonstrated a water contact angle of higher than 115° with low surface energy as well as impressive antibacterial activity. The obtained grafted cotton could be used as a promising filter screen for separating oils from contaminated water with more than 90% separation efficiency. This method is easy, environmentally friendly, cost-effective, and practical. It can be widely used to produce superhydrophobic cotton fabric on a large scale, which holds great potential in oil-water separation energy-saving clothing and healthcare products.

## Abstract 2

Multifunctional cotton fabrics with antibacterial activity, wettability, and fire resistance were fabricated. An easy, available and fast aminoalkylsilane grafting reaction was used to modify the surface of cotton fabrics.

## Graphical abstract

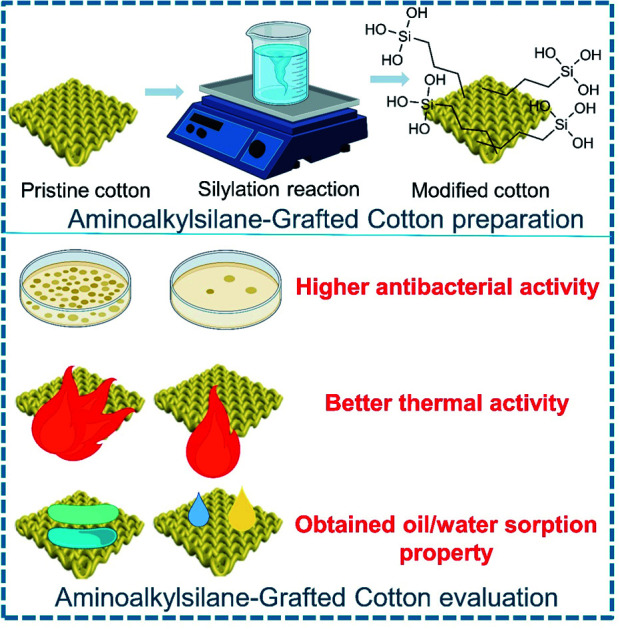

## 1. Introduction

Cotton is well known as one of the most popular resources and has been widely used in different industrial fields for many years due to its excellent properties such as biodegradability, biocompatibility, good mechanical properties, air permeability, hydrophilicity, softness, comfortableness as well as easy mass production, *etc.*^1^ Despite these properties, cotton showed some drawbacks for practical applications, such as being more combustible than most commonly available fabric, which limited the production of high-performance textile products.^2^ Besides the high natural inspiration to have non-wetting surfaces, fabrics are highly desirable to give additional protection and preserve their cleanliness against liquids and other watery contaminants.^3,4^ The other limitation is that germs could easily grow on the cotton and transmit to humans and cause sickness or even death.^5,6^

**Scheme 1:**
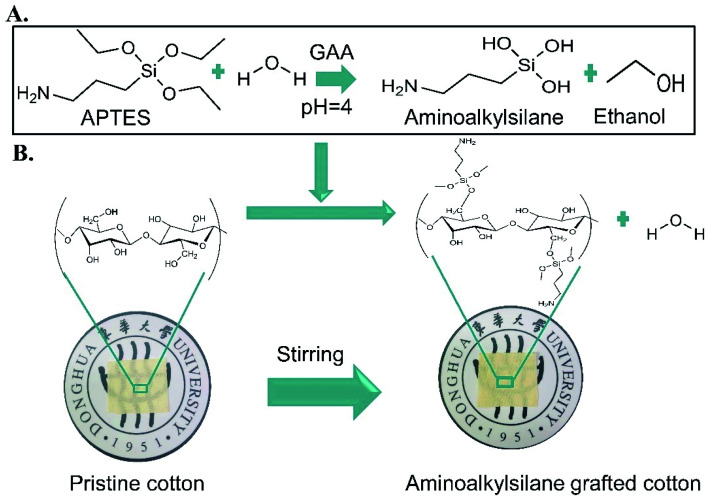
A) Hydrolysis of 3-amino propyltriethoxy silane (APTES) in the presence of glacial acetic acid (GAA) at PH = 4, (B). The chemical structure of pristine cotton and grafted cotton.

Fabrication of cotton that overcame all the limitations mentioned above would be a favourable material in various fields. Therefore, industrial and academic scientists have focused on endowing different cotton functions through fabricating and developing a versatile, low-cost, and simple preparation technology of multifunctional materials that are of great value and serve customers in other areas,^7^ such as self-cleaning, water repellent,^8^ antibacterial,^9^ anti-fogging, antifouling, anti-corrosion,^10^ oil/water separation^11^ and other applications. In order to fabricate cotton fabrics with multiple functions, many methods have been adopted, such as plasma treatments, phase separation, electrospinning methods, chemical etching, *etc.*^12^ However, these methods have disadvantages, such as costly machines, time-consuming processes, harsh conditions, complex processes, and low commercial availability.^13^ Currently, there are innovative strategies aiming to meet different requirements to change the surface properties of the fabric, such as oxidation, acetylation, sulfation, amino grafting, *etc.*^14^ Aminoalkylsilane grafting is an easy, simple, and efficient amino grafting reaction called silylation.^15^ It covalently bonds to hydroxyl (–OH) groups of pristine cotton, providing stable grafting (Scheme 1).

3-Aminopropyltrimethoxysilane (APTES) has high reactivity and is non-toxic, cheap, and commercially available, and is a simple source of aminoalkylsilane.^16^ Many studies have reported the fabrication of superhydrophobic, antibacterial, and flame-resistant cotton fabric, but none of them synthesized multifunctional cotton providing all these properties. For instance, Alongi *et al.*^17^ demonstrated a hybrid phosphorus-doped silica film grafted cotton surface through sol–gel treatment to improve only thermal and fire stability properties without evaluating wettability and antibacterial properties. While Edwards *et al.*^18^ and Xu *et al.*^19^ reported citric acid and APTES grafted cotton for antibacterial activity without studying APTES's impact on wettability or flame resistance properties. Agrawal *et al.*^6^ proposed a simple, low-cost, eco-friendly method that can enhance only the durability of superhydrophobic antibacterial cotton fabrics by using fluorine-free silane coupling agents as cross-linkers. However, the limitations of these studies were not focused on using different concentrations of APTES with varying reactions of time. Moreover, Khalaji *et al.*^16^ reported an inexpensive and simple method to construct A-*g*-BNC where aminoalkyl silane was grafted to BNC membranes and grafted through immersing membranes into APTES solutions with different concentrations. Hence, the new member showed great antibacterial activity and accelerated wound healing.

In this study, to sustain the increasing demands of modern textile manufacture, multifunctional cotton is synthesized without using hazardous solvents and by a direct, simple, and convenient method, using aminoalkylsilane grafting on the surface of cotton fabric. The present study intends to explore the relation between different reaction times and concentrations on other properties *via* grafting APTES to cotton using different concentrations and reaction times with the suggestion this method is promising for practical applications. Herein, we assume that prepared aminoalkylsilane grafted cotton fabric shows high hydrophobicity, oleophobicity, antibacterial activity, and good durability with a remarkably enhanced thermal property.

## 2. Materials and methods

### 2.1. Materials

The cotton fabric was obtained from the market, APTES (C_9_H_23_NO_3_Si, ≥99%) was purchased from Sinopharm Chemical Reagent Co, Ltd (Shanghai, China) and used without further purification. *Staphylococcus aureus* ATCC 6538 and *Escherichia coli* ATCC 25922 were obtained from American Type Culture Collection, Manassas, VA, USA.

### 2.2. Preparation, aminoalkylsilane-grafting, and reaction optimization

The cotton fabric was accurately washed with soaking in 0.2% Na_2_CO_3_ solution at 90 °C for 30 minutes in a beaker, then washed thoroughly with distilled water until neutralization was reached. After that, the fabrics were dried in an oven at 60 °C.

Aminoalkylsilane grafting was performed using the following methodology. Briefly, samples were cut into rectangle shapes with dimensions 1 cm × 2 cm and immersed in 75% (v/v) ethanol for one hour. Solutions containing different concentrations of APTES, which were 6%, 10%, and 14% (w/v) were prepared; the pH of the solution was adjusted to 4.0 ± 0.5 using glacial acetic acid (GAA) (99.5%). Cotton fabric was soaked to the APTES solutions and stirred continuously at room temperature for 6, 10, and 18 hours for each concentration (Table 1). Subsequently, the obtained samples were washed with ethanol and water and kept at 4 °C for further use.^20^

**Table 1:** The sample's name reference

Sample	Reaction time	Sample's name reference
Pristine cotton	—	C0
Pristine cotton with 6% APTES	6 h	C1-6
	10 h	C1-10
	18 h	C1-18
Pristine cotton with 10% APTES	6 h	C2-6
	10 h	C2-10
	18 h	C2-18
Pristine cotton with 14% APTES	6 h	C3-6
	10 h	C3-10
	18 h	C3-18

### 2.3. Electrochemical impedance spectroscopy

Electrochemical impedance spectroscopy (EIS) (Auto lab electrochemical workstation (PGSTAT302 N, Switzerland)) was used to detect and quantify the aminoalkylsilane content on the surface of the cotton. EIS was performed in the frequency range of 0.1 to 10^5^ Hz. An electrochemical cell with three chambers inserted the electrodes and sample holder into the solution (normal saline). The electrodes contain a reference electrode (AgCl) and an employed electrode (Pt). The ZView® software (Scribner, Southern Pines, NC) was used to find the linear fit extrapolating this interception. The following equation was used to measure the ionic conductivity:1
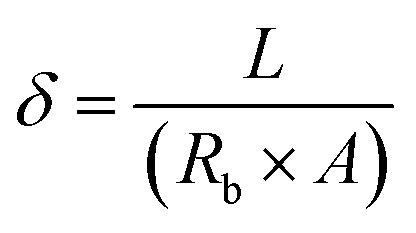
where ionic conductivity is represented *δ*, sample thickness (*L*), bulk resistance of the samples (*R*_b_), and *A* is the contact area of the samples.^21^

Ionic strength was calculated using the following equation:2
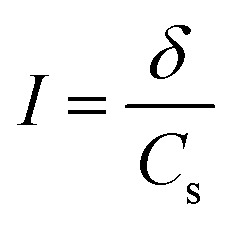
where *I* denote ionic strength *C*_s_ equal to 795 kg mS mol^−1^ cm^−1^, and *δ* represents the ionic conductivity of the samples and cotton, respectively.^22^

### 2.4. Characterization

X-ray diffraction (XRD) data of the obtained samples were measured using a XRD meter (TD-3500 Dandong Tongda Science and Technology Co, China. Cu Kα, *λ* = 1.5406 Å) with 2*θ* values in the range of 0–60°. The infrared spectra were tested using an FTIR spectrometer (Nicolet 6700, Thermo Fisher, New Jersey, U.S.A.). The spectra were recorded at the wavenumber range of 400–4000 cm^−1^ using the attenuated total reflection (ATR) technique.^23^ The test was used in 64 scans with 8 cm^−1^ resolution. The morphologies of samples were observed by scanning electron microscope (FLEX SEM1000, Hitachi, chuocho, Japan). For SEM, samples were cut and fixed on double tape, and then covered with a thin film of gold before observation. An energy dispersive spectrometer (EDS) mapping (Shimadzu, Japan) instrument attached to the SEM was used to measure the surface elemental composition.

An optical contact angle meter measured the contact angles and surface free energies (OCA15EC, Data physics, Filderstadt, Germany). In the testing process, double-sided tape attached the Sample to a glass slide, paved in the form of a plane. The surface energies of the three samples were calculated based on the Owens–Wendt–Rabel–Kaelble (OWRK) method using three different liquids: water, ethylene glycol, and ethanol. The surface tension of those liquids is given in Table 2.^23,24^ The contact angle was obtained 15 s after placing the droplets on the fabric surface.^25^

**Table 2:** Properties of liquids solution for surface energy measurement of samples

Liquid type	Surface tension (mN m^−1^)	Polar component (mN m^−1^)	Dispersion component (mN m^−1^)
Water	72.10	52.20	19.90
Ethylene glycol	48.00	29.00	19.00
Ethanol	22.10	4.60	17.50

### 2.5. Thermogravimetric analysis

The thermal stability was investigated using a thermogravimetric analyzer (TGA) (TGA 4000, PerkinElmer, New Jersey, USA). Sample masses of ∼10 mg were heated at 30–700 °C at a heating rate of 10 °C min^−1^ under nitrogen flow (20 mL min^−1^).

### 2.6. Vertical burning test

The samples' flame retardancy properties were explored using a vertical fabric flammability test (GB/T 5455). The Sample's cut edge (300 mm × 80 mm) is pendent in an enclosed chamber and exposed to a controlled methane flame for 5 seconds. The test was repeated 5 times to get reproducible data, and after flame exposure, three parameters were recorded.^26^

### 2.7. Oil/water separation test

The device was assembled by holding-prepared samples between a glass beaker and funnel to evaluate the oil/water separation process. Volume measurements demonstrated the oil separation efficiency where a certain amount of oil was poured into water in a beaker. Afterward, the oil/water mixture was poured onto the surface of the samples, and then oil was in filter samples. Separation efficiency (S) was evaluated using the following equation:

3 *S* = (*C*_a_ − *C*_b_)/*C*_a_ × 100%

where *C*_a_ is the water content in the oil/water mixture, and *C*_b_ is the collected oil after separation.^27^

### 2.8. Antibacterial activity

The direct contact method suggested by (ISO20743-2007) was used to evaluate the antibacterial activity of the aminoalkylsilane grafted cotton compared to pristine cotton. *E. coli* (Gram-negative) and *S. aureus* (Gram-positive) were used, as reported by Shahriari *et al.*^28^ Briefly, a bacterial inoculum of 0.2 mL, approximately 1 × 10^5^ to 3 × 10^5^ CFU mL^−1^ of each bacteria, was directly contacted with the sterile pieces of cotton and aminoalkylsilane grafted cotton separately then incubated at 37 °C. Following 24 h of incubation, a soya casein digest lecithin polysorbate (SCDLP) medium was used to wash out the bacterial cells under the vortex. The SCDLP medium contained casein peptone, soy peptone, potassium dihydrogen phosphate, sodium chloride, glucose, polysorbate 80, and lecithin in the amount of 17 g L^−1^, 3 g L^−1^, 2.5 g L^−1^, 5 g L^−1^, 2.5 g L^−1^, 7 g L^−1^, and 1 g L^−1^; subsequently, the pH was adjusted to 7.2 ± 0.3 using sodium hydroxide. The viable bacterial cells of both *E. coli* and *S. aureus* were diluted and spread on the soled agar plate to count the colonies. Each sample had three replications, technically and biologically. The antibacterial mechanism of cotton and synthesized materials was investigated by observing the bacteria *via* SEM. Briefly, bacterial culture of 0.1 mL containing approximately 10^5^ CFU mL^−1^ was directly contacted with a sterile piece of cotton and aminoalkylsilane grafted cotton, followed by incubation at 37 °C for 24 h. Subsequently, the bacterial cells were fixed on the surface of the materials using 1 mL of 2.5% glutaraldehyde and incubated for 4 h with light protection. Then, normal saline was used to wash the materials three times, and ethanol gradients of 25%, 50%, 75%, 90%, and 100% were employed to dehydrate the materials. Each ethanol concentration was contacted with the samples for 15 min. Ethanol was replaced with tertiary butanol and incubated for another 20 min, followed by freeze-drying. The bacteria on each Sample's surface were observed using SEM (FEI Quanta FEG 250).

## 3. Results and discussion

### 3.1. Electrochemical characterization

**Fig. 1:**
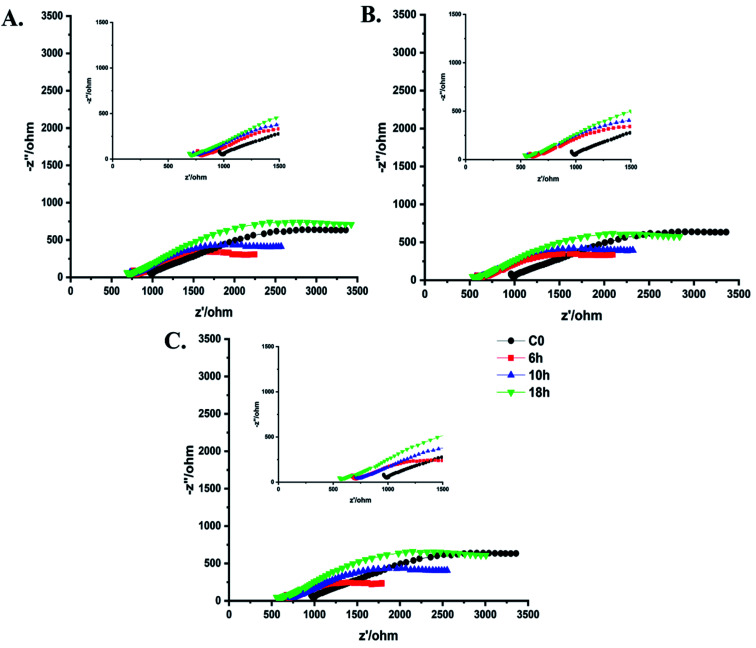
Electrochemical impedance spectra (EIS) of pristine cotton and aminoalkylsilane grafted cotton with different reaction times and APTES concentrations of (A) 6%. (B) 10%. (C) 14%.

Electrochemical Impedance Spectroscopy (EIS) for pristine cotton and aminoalkylsilane grafted cotton has been conducted in case to evaluate the effect of different APTES concentrations and reaction times on aminoalkylsilane grafting content. The obtained data from using ZView® software is most frequently plotted by the Nyquist plot (−Z′′ *vs* Z′) to express ionic conductivity and ionic strength calculation, as seen in Fig. 1.^29^Table 3 reveals the importance of APTES concentration and reaction time on aminoalkylsilane grafted cotton. The successfully grafting of aminoalkylsilane for all samples was seen because their higher ionic conductivity than pristine cotton (0.72 mS cm^−1^ corresponding to bulk resistance of 1838 Ω cm^2^) was remarkably increased after increased reaction time. After 6 hours of reaction time the ionic conductivity was 2.64 mS cm^−1^, 4.07 mS cm^−1^, 6.28 mS cm^−1^ corresponding to bulk resistance 1038 Ω cm^2^, 650 Ω cm^2^, 608 Ω cm^2^ and ionic strength 0.0156 mol kg^−1^, 0.0243 mol kg^−1^, 0.0338 mol kg^−1^ for C1-6, C2-6, C3-6, respectively. These results agreed with the discussion above.

**Table 3:** Bulk resistance, ionic conductivity, and ionic strength of pristine cotton and aminoalkylsilane grafted cotton with different reaction times and APTES concentration

Material	Bulk resistance (ohm)	Ionic conductivity (mS cm^−1^)	Ionic strength (mol kg^−1^)
C0	1838	0.72	0.0008
C1-6	1038	2.64	0.0156
C1-10	1000	2.96	0.0176
C1-18	1059	2.31	0.0134
C2-6	650	4.07	0.0243
C2-10	605	5.93	0.0321
C2-18	875	4.65	0.0297
C3-6	608	6.18	0.0338
C3-10	578	7.35	0.0414
C3-18	613	6.03	0.0254

Moreover, it was clear that using the same concentration of APTES ionic conduction was enhanced by increasing the reaction time till 10 h. Still, with reach reaction time up to 18 hours, there was a significant decline in ionic conductivity, bulk resistance, and ionic strength values, respectively, as listed in Table 3. Interestingly, the graft of aminoalkylsilane was effectively increased with increasing APTES concentration in each time point. That proves that the APTES concentration and reaction time play a significant role in the aminoalkylsilane grafting reaction. It is well-known that the highest electronic and ionic conduction during cycling were registered at 10 hours of reaction time for all APTES concentrations, which were 2.96, 5.93, and 7.35 mS cm^−1^ for 1000, 605, and 578 Ω cm^2^ bulk resistance for C1-10, C2-10, and C3-10, respectively. APTES grafted cotton was further prepared using 18% APTES for 10 h reaction time (C3-10) to achieve superior ionic conductivity.

### 3.2. Chemical structure analysis

**Fig. 2:**
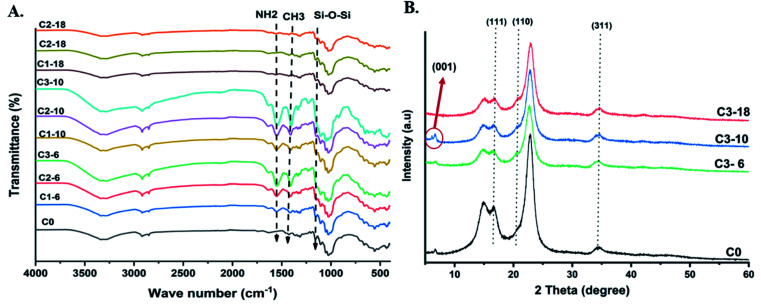
A) FTIR spectra of pristine cotton and aminoalkylsilane grafted cotton with different reaction times. (B) XRD diffraction test for pristine cotton and aminoalkylsilane grafted cotton.

The chemical structure of pristine cotton and aminoalkylsilane grafted cotton were studied *via* FTIR spectra (4000–400 cm^−1^), as shown in Fig. 2(A). It was observed that several strong peaks for pure cotton had been illustrated at 3335 cm^−1^, 2900 cm^−1^, and 1105 cm^−1^ represented –OH, C–H, and C–O–C glycosidic bond, respectively.^30^

Furthermore, the peaks related to aminoalkylsilane grafted were seen at 1601 cm^−1^ and 1479 cm^−1^, which attributed to the amine (NH_2_) bond deformation and the CH_2_ deformation vibration, respectively.^31^ Meanwhile, peaks at 1566 cm^−1^ 1484 cm^−1^ correspond to the NH_3_ deformation due to hydrogen bonding. At the same time, peaks at 1387 cm^−1^ correspond to symmetric deformation of the CH_3_ groups. A new peak at 1636 cm^−1^ was observed and represented corresponds to C=C bond. APTES treated cotton's chemical structure observed the similarity between pristine cotton and aminoalkylsilane grafted cotton.^17^ In addition, the peak for the primary amino group (NH_2_), which is present in aminoalkylsilane, was shown at 1560 cm^−1^. Peaks at 1200 cm^−1^–1100 cm^−1^ have been reported to Si–O–Si and Si–O–, respectively. Thus, the strong peaks arise from cotton's C–O–C vibrations.^19^

Fig. 2(B) shows the XRD patterns of pristine cotton and aminoalkylsilane grafted cotton (14%) at 6 hours, 10 hours, and18 hours. Notably, it was clear that are peaks for all curves are very close to each other. Diffraction peaks at 16.75°, 21°, and 34.89° were indexed to 111, 110, 311, respectively. Moreover, the XRD result revealed a peak cantering at 22° of 2*θ* was observed corresponding to silica at APTES. In addition, another diffraction peak at 5.71° for Sample C3-10 has been strongly seen, which was corresponded to (001), which is attributed to the intercalation of APTES. However, upon comparing the diffraction patterns curves of aminoalkylsilane grafting cotton, they showed a slight decrease in peak intensity.^32^

### 3.3. Surface morphology analysis

**Fig. 3:**
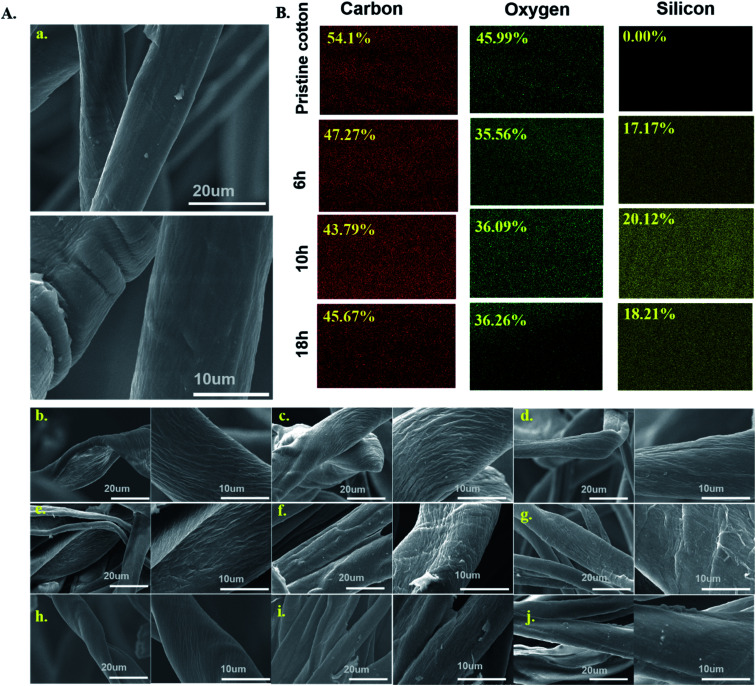
(A). SEM (a) pristine cotton. (b) C1-6. (c) C1-10. (d) C1-18. (e) C2-6. (f) C2-10. (g) C2-18. (h) C3-6. (i) C3-10. (j) C3-18 (B) EDS mapping of pristine cotton and C3 with variation reaction time.

The scanning electron microscopy images for pristine cotton and aminoalkylsilane grafted cotton with different concentrations were illustrated in Fig. 3(A). Pristine cotton fabric visually has a smooth microscopic surface with characteristic parallel ridges and grooves composed of winding and thin cotton fibres, as shown in Fig. 3(A(a)). It is worth noticing that after the modification of cotton with APTES, the surface has changed dramatically, and there are different features on the surface. The surface of the aminoalkylsilane grafted cotton showed a striped and more distinct microfibrillar structure (twisted and wrinkled surfaces), which could enhance the roughness of the surfaces, indicating that the cotton is chemically bonded with the aminoalkylsilane grafted, which is well coupled with the low surface energy of aminoalkylsilane grafted to display superhydrophobic material.^6,33^

However, when the concentration of APTES increased from 6% to 10%, the surface of cotton became rougher. Hence, the structure of the cotton fiber is partially destroyed, which promotes the adsorption and permeation inside the fibre. It was observed that surface roughness increased with up to 10% filler loading, as seen in Fig. 3(A(c, f and i)) but remarkably decreased at 18%, as seen in Fig. 3(A(d, g and j)), resulting in lower hydrophobicity.

Investigating surface elements is an important technique to demonstrate the surface chemical composition changes directly. EDS's elemental mapping scanning mode is a qualitative analysis used to examine the chemical composition, and the results are shown in Fig. 3(B). It was observed that there are only C and O elements in the pristine cotton. At the same time, it is noteworthy that aminoalkylsilane grafted cotton shows the presence of Si elements besides C and O, respectively, as reported in figure. EDS mapping results indicating silicon elements were 0%, 17.17%, 20.12% and 18.21% for C0, C3-6, C3-10, and C3-18, respectively. This finding proved that SI the element was introduced into cellulose, consistent with the results obtained from FTIR and XRD.

### 3.4. Wettability properties analysis

The surface wettability property is closely related to the surface chemical composition. The pristine cotton confirmed the super hydrophilic property, which could adsorb various water-based droplets. At the same time, the grafted cotton with different ratios of APTES could be repelled by these liquids and form a completely round shape, as seen in Fig. 3(A(a)). This behaviour indicates that aminoalkylsilane grafted has excellent superhydrophobicity properties. The interface phenomena confirm that aminoalkylsilane grafted is hydrophobic and super oleophilic, appropriate for separating the oil/water mixture.

It was observed from Table 4 and Fig. 3(A(b)) that samples registered the highest water contact angle with different concentrations at reaction time 10 hours was 132.8°, 141.45°, 155.52° for C1-10, C2-10, C3-10, followed by grafted samples with reaction time 18 hours and 6 hours, respectively. This behaviour was confirmed with SEM and chemical analysis; the hierarchical structures were formed to enhance the roughness further. However, the roughness becomes less while reaching 18 hours, causing a decline in the water contact angles, as seen in figure. It is worth noting that using the same reaction time with different concentrations for all grafted samples increased the water contact angles with further APTES ratio and enhanced surface roughness. These consequences indicated that the optimal concentration for preparing aminoalkylsilane grafted cotton based on proper reaction time was in the range of 10 hours. The surface energies of cotton treated with different concentrations of APTES, their measured water contact angles, ethylene glycol, and ethanol is calculated and presented in Table 2. Aminoalkylsilane grafted showed very low surface energy below 52 mN m^−1^. This was close to the surface energy of oils (20–30 mN m^−1^) and far below that of water (72 mN m^−1^),^34^ contributing to the fabric superhydrophobicity, with a water contact angle up to 118°. Therefore, oil droplets displaced water droplets, while water repelled outside the surface due to having high contact angles, which are substantially spherical in shape, and a high energy barrier.^34,35^

**Table 4:** Surface free energy of pristine cotton and aminoalkylsilane grafted cotton

Materials	Contact angle (°)			Surface energy (mN m^−1^)
	Water	Ethylene glycol	Ethanol	
C0		—	—	—
C1-6	118.32	68.78	32.32	52.67
C1-10	132.80	89.34	34.18	48.95
C1-18	125.72	77.94	30.14	50.38
C2-6	127.45	80.45	35.67	47.94
C2-10	141.45	104	37.89	43.68
C2-18	134.78	88.18	35.89	46.45
C3-6	136.83	99.13	38.90	44.32
C3-10	155.52	117.53	42.13	38.55
C3-18	139.67	98.23	40.13	43.78

**Fig. 4:**
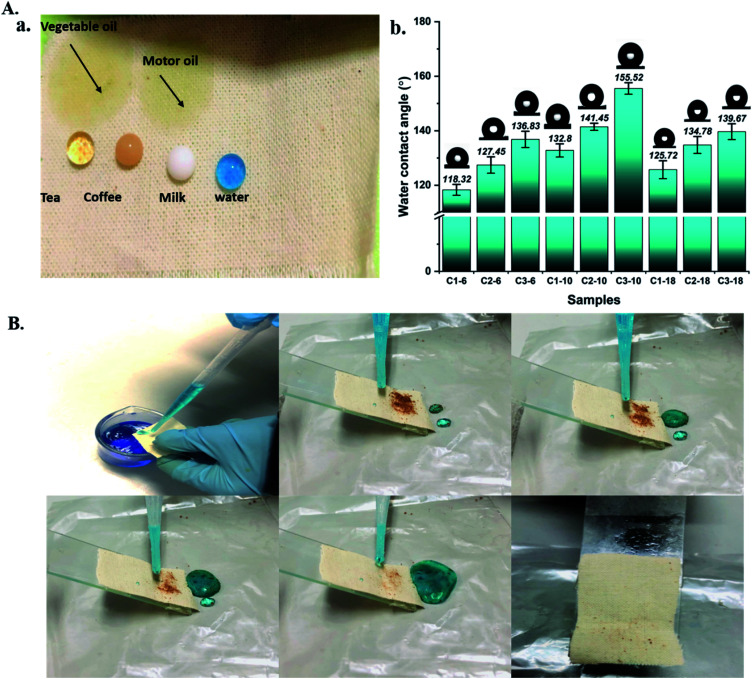
(A) (a) Digital images of liquid drops on aminoalkylsilane grafted cotton, (b) water contact angles of aminoalkylsilane grafted cotton. (B) The self-cleaning process of superhydrophobic surfaces with low surface energy.

The self-cleaning ability of hydrophobic materials is one of the essential properties for their real application. This study used coffee as a contamination material to evaluate the grafted cotton fabric's self-cleaning performance, as seen in Fig. 4(B). Aminoalkylsilane grafted cotton fabric with a high static contact angle showed a high self-cleaning ability where coffee was removed and cleaned by rinsing with water. Similar findings have also been reported recently in the literature.^6,36^ Conversely, coffee could not be carried out by water where it was wetted and adhered to the surface of pristine cotton.

### 3.5. Oil–water separation

**Fig. 5:**
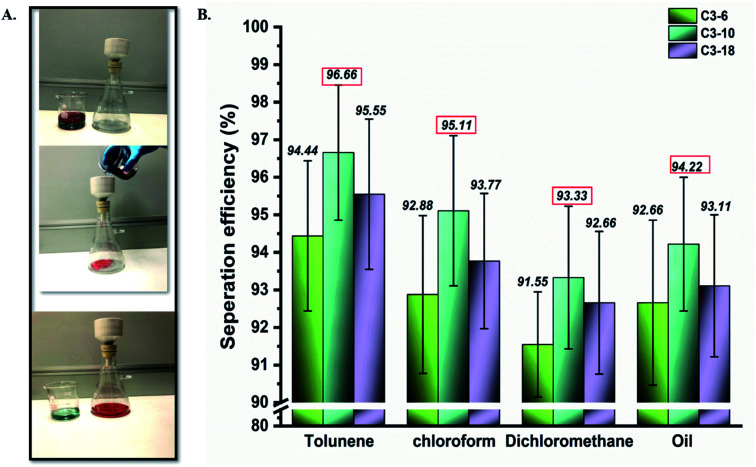
(A) Applications in oil/water separation. (a) Oil–water Separation with grafted cotton fabric using a mixture of water (dyed blue) and soybean oil (dyed red) was poured into the separating facility. (B)The separation efficiency of different kinds of oil/water mixtures.

The hydrophobic behaviour of aminoalkylsilane grafted cotton, as mentioned above, could extend its practical application to remove oil from oily wastewater mixtures.^37^ The separation performance of grafted cotton with APTES was studied, where it could separate the oil-organic liquid and water from their mixture by gravitation.^3,38,39^ As shown in Fig. 5(A)(a) sequence of mixtures, including toluene/water, dichloromethane/water, and chloroform/water, were prepared in this study. Hence, Grafted cotton fabric with high hydrophobicity was firmed between the funnel and conical flask, as shown in Fig. 5. A mixture with 50 mL water and 50 mL soybean oil was chosen as the samples and trickled into the separation device. A liquid mixture containing water and soybean oil was dyed with methyl blue and Sudan III.^39^ The phenomenon of oil–water separation was amazingly observed. Due to the superoleophobicity and hydrophobicity of the grafted cotton fabric, water was repelled while oil quickly penetrated through the fabric.

Moreover, the highest oil separation was registered for samples with a reaction time of up to 10 hours, followed by 18 and 6 hours. As exhibited in Fig. 5(B), the highest separation efficiencies noted by sample C3-10 for toluene, chloroform, dichloromethane, and oil with expected values were 95.66%, 95.11%, 93.33%, 94.22%, respectively.

### 3.6. Thermal properties

**Fig. 6:**
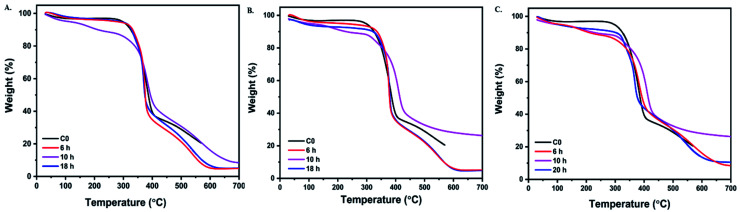
TGA curves of pristine cotton and aminoalkylsilane grafted cotton at different reaction times (A) 6%. (B) 10%. (C) 14%.

The thermal stability of cotton before and after grafting was investigated using thermal gravity analysis under nitrogen atmosphere (TGA). The typical curves of pristine cotton and its graft are plotted in Fig. 6. Some essential data from TGA, such as Tonset degradation temperature, maximum-rate degradation temperature (*T*_max_), and char residual amount at 500 °C and 600 °C, are listed in Table 5. The low weight loss was noticed under 150 °C, which was caused by the evaporation of the moister inside fabric. Fig. 6(A–C) shows that only two steps register the thermal degradation of pristine cotton under nitrogen conditions. It started to decompose at a registered degree 340 °C, involving the decomposition of the glycosyl units to char and depolymerizing of such units to volatile species at a higher temperature.^40^

**Table 5:** TGA results of pristine cotton and aminoalkylsilane grafted cotton with different reaction times

Material	Tonset	T1	T2	R500	R600
C0	340	416.37	—	29.07	—
C1-6	364.06	404.74	597	20.31	5.19
C1-10	360.13	422.78	—	31.89	15.99
C1-18	342.71	388.54	607	23.34	6.15
C2-6	367.12	406.90	611.71	22.67	6.11
C2-10	385.31	436.22	—	33.45	28.13
C2-18	350.13	394.34	609.34	23.88	7.12
C3-6	369.81	426.27	625.67	24.08	16.14
C3-10	389.12	455	—	38.90	31.12
C3-18	341.90	388.56	623	30.52	13.34

There is only char residue at 500 °C (29.07%), while at 600 °C (0%), there is no char residue.^41^ While the presence of APTES displays an essential role in enhancing thermal stability; as demonstrated in Fig. 6(C), it was noticed that Tonset degradation temperature increased gradually compared with pristine cotton, which was 369.81 °C, 389.12 °C, 341.90 °C, 340 °C for C3-6, C3-10, C3-18, and C0, respectively. After the decomposition process, it is evident that there was far more char residual left of the grafted cotton measured at 600 °C than of pristine cotton, where the retained residues were 16.14%,31.12%, 13.34%, 0% for C3-6, C3-10, C3-18, and C0, respectively. Moreover, it was observed that with using the different concentration with respect time the temperature T2 increase as we can see from Table 5 samples follow the trend: C3-18 > C2-18 > C1-18.

These results indicated that aminoalkylsilane could change the pyrolytic process of the cotton fibers to form char efficiently. Aminoalkylsilane grafted cotton showed increased thermal stability by shifting T2 to higher temperatures, as shown in Table 4. This could be explained by thermal insulation done by silica, where the silica can behave as a physical, thermal barrier.

### 3.7. Flame retardancy

**Fig. 7:**
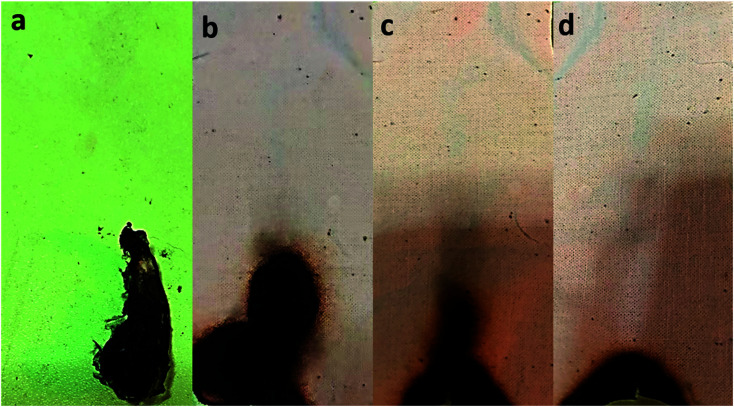
Digital images of the pristine cotton and aminoalkylsilane grafted cotton after vertical testing (a) C0. (b) C1-10. (c) C2-10. (d). C3-10.

A vertical burning test examined the flame retardancy of the pristine cotton and aminoalkylsilane grafted in terms of flammability. The flammability data such as after flame, afterglow, and char length are listed in Table 6. It was observed that pristine cotton ignited immediately and burned out with 22 s, and there was only ash formed. While the aminoalkylsilane grafted cotton after 12 s of ignition showed narrow chars at the bottom of cotton samples with different char lengths as listed in Table 6 and shown in Fig. 7.

**Table 6:** Collected data of pristine cotton and aminoalkylsilane grafted cotton after flame test

Material	After flame time (s)	Afterglow time (s)	Char length (mm)
C0	15	12	0
C1-6	0	0	13.50
C1-10	0	0	8.50
C1-18	0	0	12.40
C2-6	0	0	11.80
C2-10	0	0	7.30
C2-18	0	0	11.40
C3-6	0	0	10
C3-10	0	0	5.50
C3-18	0	0	9.80

The results of the vertical flammability test are demonstrated in Fig. 7. It can be seen that char lengths of aminoalkylsilane grafted cotton become less with the increase in the concentration of APTES concerning time (6 hours). For instance, the samples follow the C3-6 < C2-6 < C1-6. While, char lengths of aminoalkylsilane grafted cotton decrease with the increased reaction time up to 10 hours for the same concentration then increase when reaching a reaction time 18 hours. Moreover, at a reaction time 10 hours using the same APTES concentration, the char length was less than other samples where char length was 8.5 mm, 7.3 mm, 5.5 mm for C1-10, C2-10, C3-10, respectively. It was observed that aminoalkylsilane grafted cotton reduces the burning time and protects samples from flame. This behaviour is explained due to the presence of silica element inside APTES, which acts as a protection against fire.

### 3.8. Antibacterial activity

The antibacterial activities of pristine cotton and aminoalkylsilane grafted cotton against *E. coli* and *S. aureus* were evaluated using the direct contact method. The results indicated that the antibacterial activity of the aminoalkylsilane grafted cotton is impressively improved compared to pristine cotton, which is attributed to aminoalkylsilane on the surface of synthesized materials. The antibacterial activity against both bacterial strains was significantly enhanced compared to pristine cotton (higher than 5 log values). The result indicated that antibacterial activity was enhanced following APTES concentration enhancement.

**Fig. 8:**
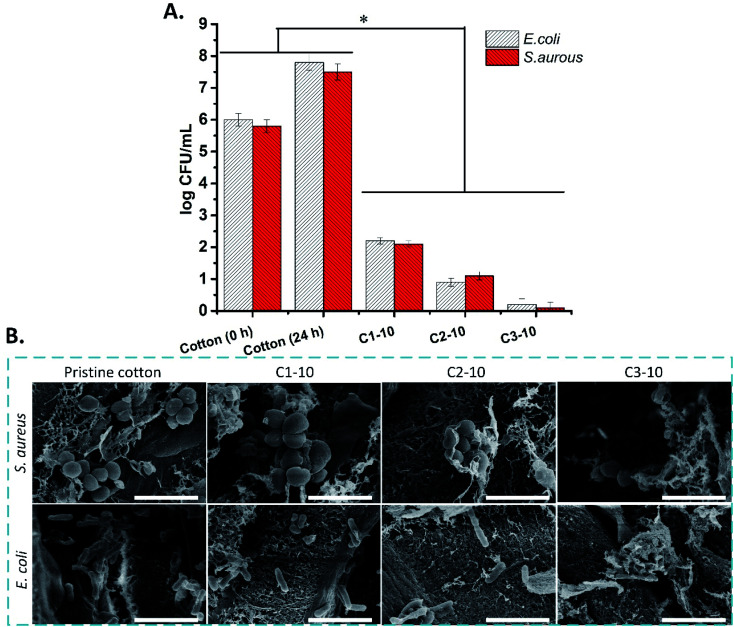
(A) Antibacterial activity of pristine cotton after 0 and 24 h in comparison to aminoalkane saline grafted cotton with the concentration of 6, 10, and 14 (w/v) after 24 h of direct contact with *E. coli* and *S. aureus.* * (*p* ≤ 0.05). (B) SEM image of *E. coli* and *S. aureus* after 24 h direct contact with pristine cotton and aminoalkane saline grafted cotton (scale bar = 2 μm).

The pristine cotton and aminoalkane saline grafted cotton surface was observed after 24 h of direct contact with *E. coli* and *S. aureus*. The bacteria on the surface of pristine cotton indicated a smooth wall without any damage. However, the bacteria on the aminoalkanesaline grafted cotton surface had damaged the cell wall. Additionally, all the aminoalkane saline grafted cotton indicated antibacterial activity by cell wall damage. Aminoalkylsilane grafted cotton also inhibited the growth of the bacteria, as it can be seen that a greater number of the bacteria is present on the pristine cotton than aminoalkane saline grafted cotton (Fig. 8B).

## 4. Conclusion

In this work, we report a versatile approach has been developed to design a successful fabrication of multifunctional fabrics through the chemical grafting of aminoalkylsilane. Electrochemical impedance spectroscopy (EIS) confirmed the correlation between reaction time and APTES concentration. FTIR and XRD analysis indicated the successful grafting of APTES on the cotton surface. SEM morphological analysis revealed that APTES enhances the surface roughness of cotton surfaces. The result indicated that 10 hours of reaction time with the APTES concentrations of 14% provide the highest aminoalkylsilane grafting on cotton's surface. The obtained aminoalkylsilane grafted cotton showed high hydrophobicity with a water contact angle of more than 115° for all samples, while the highest water contact angle with different concentrations, registered at a reaction time of 10 hours, was more than 130°; besides, it showed enhancement of the self-cleaning ability. The aminoalkylsilane grafted cotton selectively extracted oil from different types of oil/water mixtures with high separation efficiency (>90%). It also provided impressive antibacterial activity by damaging both *E. coli* and *S. aureus* cell walls. This study reports an environmentally friendly, cost-effective strategy that could display tremendous potential for large-scale production.

## Conflicts of interest

The authors declare no conflict of interest.

## Supplementary Material

## References

[cit1] Zouari R., Gargoubi S. (2021). Enhancing Flame Resistance of Cellulosic Fibers Using an Ecofriendly Coating. Coatings.

[cit2] Liu Y., Pan Y.-T., Wang X., Acuña P., Zhu P., Wagenknecht U., Heinrich G., Zhang X.-Q., Wang R., Wang D.-Y. (2016). Effect of phosphorus-containing inorganic–organic hybrid coating on the flammability of cotton fabrics: Synthesis, characterization and flammability. Chem. Eng. J..

[cit3] Deng Y., Han D., Deng Y.-Y., Zhang Q., Chen F., Fu Q. (2020). Facile one-step preparation of robust hydrophobic cotton fabrics by covalent bonding polyhedral oligomeric silsesquioxane for ultrafast oil/water separation. Chem. Eng. J..

[cit4] Moiz A., Padhye R., Wang X. (2018). Durable superomniphobic surface on cotton fabrics *via* coating of silicone rubber and fluoropolymers. Coatings.

[cit5] Qiang S., Chen K., Yin Y., Wang C. (2017). Robust UV-cured superhydrophobic cotton fabric surfaces with self-healing ability. Mater. Des..

[cit6] Agrawal N., Low P. S., Tan J. S. J., Fong E. W. M., Lai Y., Chen Z. (2020). Durable easy-cleaning and antibacterial cotton fabrics using fluorine-free silane coupling agents and CuO nanoparticles. Nano Mater. Sci..

[cit7] Bao W., Liang D., Zhang M., Jiao Y., Wang L., Cai L., Li J. (2017). Durable, high conductivity, superhydrophobicity bamboo timber surface for nanoimprint stamps. Prog. Nat. Sci.: Mater. Int..

[cit8] Pan G., Xiao X., Yu N., Ye Z. (2018). Fabrication of superhydrophobic coatings on cotton fabric using ultrasound-assisted in-situ growth method. Prog. Org. Coat..

[cit9] Ankudze B., Asare B., Goffart S., Koistinen A., Nuutinen T., Matikainen A., Andoh S. S., Roussey M., Pakkanen T. T. (2019). Hydraulically pressed silver nanowire-cotton fibers as an active platform for filtering and surface-enhanced Raman scattering detection of bacteria from fluid. Appl. Surf. Sci..

[cit10] Islam S. R., Patoary M. K., Farooq A., Naveed T., Ahmed K., Shao H., Jiang J. (2022). 3D Weft-knitted spacer fabrics (WKSFs) coated with silica aerogels as oil intercepting sorbents for use in static and dynamic water tests. Ind. Crops Prod..

[cit11] Wang W., Liu R., Chi H., Zhang T., Xu Z., Zhao Y. (2019). Durable superamphiphobic and photocatalytic fabrics: tackling the loss of super-non-wettability due to surface organic contamination. ACS Appl. Mater. Interfaces.

[cit12] Caschera D., Mezzi A., Cerri L., de Caro T., Riccucci C., Ingo G. M., Padeletti G., Biasiucci M., Gigli G., Cortese B. (2014). Effects of plasma treatments for improving extreme wettability behavior of cotton fabrics. Cellulose.

[cit13] Shabanian S., Khatir B., Nisar A., Golovin K. (2020). Rational design of perfluorocarbon-free oleophobic textiles. Nature Sustainability.

[cit14] Song W., Zeng Q., Yin X., Zhu L., Gong T., Pan C. (2018). Preparation and anticoagulant properties of heparin-like electrospun membranes from carboxymethyl chitosan and bacterial cellulose sulfate. Int. J. Biol. Macromol..

[cit15] Monasterio F. E., Dias M. L., Pita V. J., Erdmann E., Destefanis H. A. (2010). Effect of the organic groups of difunctional silanes on the preparation of coated clays for olefin polymer modification. Clay Miner..

[cit16] Shahriari-Khalaji M., Hu G., Chen L., Cao Z., Andreeva T., Xiong X., Krastev R., Hong F. F. (2021). Functionalization of Aminoalkylsilane-Grafted Bacterial Nanocellulose with ZnO-NPs-Doped Pullulan Electrospun Nanofibers for Multifunctional Wound Dressing. ACS Biomater. Sci. Eng..

[cit17] Alongi J., Colleoni C., Rosace G., Malucelli G. (2012). Thermal and fire stability of cotton fabrics coated with hybrid phosphorus-doped silica films. J. Therm. Anal. Calorim..

[cit18] Edwards J. V., Prevost N. T., Condon B., French A. (2011). Covalent attachment of lysozyme to cotton/cellulose materials: protein verses solid support activation. Cellulose.

[cit19] Xu A. Y., McGillivray D. J., Dingley A. J. (2021). Active antibacterial coating of cotton fabrics with antimicrobial proteins. Cellulose.

[cit20] Shahriari-Khalaji M., Li G., Liu L., Sattar M., Chen L., Zhong C., Hong F. F. (2022). A poly-l-lysine-bonded TEMPO-oxidized bacterial nanocellulose-based antibacterial dressing for infected wound treatment. Carbohydr. Polym..

[cit21] Huang C., Ji H., Yang Y., Guo B., Luo L., Meng Z., Fan L., Xu J. (2020). TEMPO-oxidized bacterial cellulose nanofiber membranes as high-performance separators for lithium-ion batteries. Carbohydr. Polym..

[cit22] Foustoukos D. I. (2016). On the ionic strength and electrical conductivity of crustal brines. Chem. Geol..

[cit23] Islam S. R., Alassod A., Naveed T., Dawit H., Ahmed K., Jiang J. (2021). The study of hydrophobicity and oleophilicity of 3D weft-knitted spacer fabrics integrated with silica aerogels. J. Ind. Text..

[cit24] Cao S., Dong T., Xu G., Wang F. (2016). Study on structure and wetting characteristic of cattail fibers as natural materials for oil sorption. Environ. Technol..

[cit25] Alassod A., Abedalwafa M. A., Xu G. (2021). Evaluation of polypropylene melt blown nonwoven as the interceptor for oil. Environ. Technol..

[cit26] Jang Y.-M., Yu C.-J., Choe K.-S., Choe C.-H., Kim C.-H. (2021). Preparation and flame retardant properties of cotton fabrics treated with resorcinol bis (diphenyl phosphate). Cellulose.

[cit27] Shang Q., Hu L., Yang X., Hu Y., Bo C., Pan Z., Ren X., Liu C., Zhou Y. (2021). Superhydrophobic cotton fabric coated with tannic acid/polyhedral oligomeric silsesquioxane for highly effective oil/water separation. Prog. Org. Coat..

[cit28] Shahriari-Khalaji M., Hong S., Hu G., Ji Y., Hong F. F. (2020). Bacterial Nanocellulose-Enhanced Alginate Double-Network Hydrogels Cross-Linked with Six Metal Cations for Antibacterial Wound Dressing. Polymers.

[cit29] Sriram B., Baby J. N., Wang S.-F., Govindasamy M., George M., Jothiramalingam R. (2020). Cobalt molybdate nanorods decorated on boron-doped graphitic carbon nitride sheets for electrochemical sensing of furazolidone. Microchim. Acta.

[cit30] Yue Y., Han J., Han G., Zhang Q., French A. D., Wu Q. (2015). Characterization of cellulose I/II hybrid fibers isolated from energycane bagasse during the delignification process: morphology, crystallinity and percentage estimation. Carbohydr. Polym..

[cit31] Rao X., Abou Hassan A., Guyon C., Zhang M., Ognier S., Tatoulian M. (2020). Plasma polymer layers with primary amino groups for immobilization of nano-and microparticles. Plasma Chem. Plasma Process..

[cit32] Abeywardena S. B., Perera S., Nalin de Silva K., Tissera N. P. (2017). A facile method to modify bentonite nanoclay with silane. Int. Nano Lett..

[cit33] (a) Azizian S. and Khosravi M., Advanced oil spill decontamination techniques, in Interface Science and Technology, vol. 30, Elsevier, 2019; pp. 283–332

[cit34] Islam S. R., Yu W., Naveed T. (2019). Influence of silica aerogels on fabric structural feature for thermal isolation properties of weft-knitted spacer fabrics. J. Eng. Fibers Fabr..

[cit35] Dong T., Wang F., Xu G. (2015). Sorption kinetics and mechanism of various oils into kapok assembly. Mar. Pollut. Bull..

[cit36] Yang M., Liu W., Liang L., Jiang C., Liu C., Xie Y., Shi H., Zhang F., Pi K. (2020). A mild strategy to construct superhydrophobic cotton with dual self-cleaning and oil–water separation abilities based on TiO_2_ and POSS *via* thiol-ene click reaction. Cellulose.

[cit37] Pan Y., Huang S., Li F., Zhao X., Wang W. (2018). Coexistence of superhydrophilicity and superoleophobicity: Theory, experiments and applications in oil/water separation. J. Mater. Chem. A.

[cit38] Li L., Rong L., Xu Z., Wang B., Feng X., Mao Z., Xu H., Yuan J., Liu S., Sui X. (2020). Cellulosic sponges with pH responsive wettability for efficient oil-water separation. Carbohydr. Polym..

[cit39] Alassod A., Khalaji M. S., Islam S. R., Xu G., Chaudary A., Alam M., Alkhateeb W. (2022). Polypropylene-chitosan sponges prepared *via* thermal induce phase separation used as sorbents for oil spills cleanup. Polym. Bull..

[cit40] Wang N., Liu Y., Liu Y., Wang Q. (2017). Properties and mechanisms of different guanidine flame retardant wood pulp paper. J. Anal. Appl. Pyrolysis.

[cit41] Xue C.-H., Zhang L., Wei P., Jia S.-T. (2016). Fabrication of superhydrophobic cotton textiles with flame retardancy. Cellulose.

